# Temporal relationship between haemodynamic changes and activation of closed-loop stimulation during a tilt-induced vasovagal syncope

**DOI:** 10.1093/europace/euae045

**Published:** 2024-02-10

**Authors:** Vincenzo Russo, Marco Tomaino, Erika Parente, Angelo Comune, Daniele Giacopelli, Paola Napoli, Alessio Gargaro, Michele Brignole

**Affiliations:** Cardiology and Syncope Unit, Department of Translational Medical Sciences, University of Campania ‘Luigi Vanvitelli’—Monaldi Hospital, 80126 Naples, Italy; Department of Cardiology, Ospedale Generale Regionale, Bolzano, Italy; Cardiology and Syncope Unit, Department of Translational Medical Sciences, University of Campania ‘Luigi Vanvitelli’—Monaldi Hospital, 80126 Naples, Italy; Cardiology and Syncope Unit, Department of Translational Medical Sciences, University of Campania ‘Luigi Vanvitelli’—Monaldi Hospital, 80126 Naples, Italy; Research Clinical Unit, Biotronik Italy, Milan, Italy; Research Clinical Unit, Biotronik Italy, Milan, Italy; Research Clinical Unit, Biotronik Italy, Milan, Italy; IRCCS Istituto Auxologico Italiano, Faint and Fall Research Centre, Department of Cardiology, S. Luca Hospital, Piazzale Brescia 20, 20149 Milan, Italy

**Keywords:** Vasovagal syncope, Tilt table test, Pacemaker therapy, Close-loop stimulation

## Abstract

**Aims:**

A dual-chamber pacemaker with closed-loop stimulation (CLS) mode is effective in reducing syncopal recurrences in patients with asystolic vasovagal syncope (VVS). In this study, we explored the haemodynamic and temporal relationship of CLS during a tilt-induced vasovagal reflex.

**Methods and results:**

Twenty patients underwent a tilt test under video recording 3.9 years after CLS pacemaker implantation. Three patients were excluded from the analysis because of no VVS induced by the tilt test (*n* = 1) and protocol violation (*n* = 2). In 14 of the remaining 17 patients, CLS pacing emerged during the pre-syncopal phase of circulatory instability when the mean intrinsic heart rate (HR) was 88 ± 12 b.p.m. and systolic blood pressure (SBP) was 108 ± 19 mmHg. The CLS pacing rate thereafter rapidly increased to 105 ± 14 b.p.m. within a median of 0.1 min [inter-quartile range (IQR), 0.1–0.7 min] when the SBP was 99 ± 21 mmHg. At the time of maximum vasovagal effect (syncope or pre-syncope), SBP was 63 ± 17 mmHg and the CLS rate was 95 ± 13 b.p.m. The onset of CLS pacing was 1.7 min (IQR, 1.5–3.4) before syncope or lowest SBP. The total duration of CLS pacing was 5.0 min (IQR, 3.3–8.3). Closed-loop stimulation pacing was not observed in three patients who had a similar SBP decrease from 142 ± 22 mmHg at baseline to 69 ± 4 mmHg at the time of maximum vasovagal effect, but there was no significant increase in HR (59 ± 1 b.p.m.).

**Conclusion:**

The reproducibility of a vasovagal reflex was high. High-rate CLS pacing was observed early during the pre-syncopal phase in most patients and persisted, although attenuated, at the time of maximum vasovagal effect.

**Registration:**

ClinicalTrials.gov identifier: NCT06038708

## Graphical Abstract

**Graphical Abstract:**
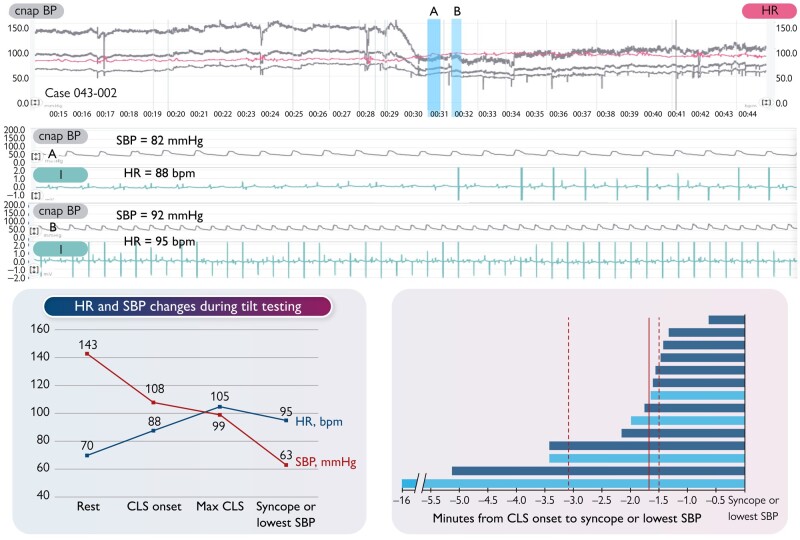

**What’s new?**

- We examined the temporal relationship between haemodynamic changes and closed-loop stimulation (CLS) pacing mode during a tilt-induced vasovagal reflex.
- The onset of CLS pacing was 1.7 min (inter-quartile range, 1.5–3.4) before the maximum vasovagal effect.
- The maximum CLS rate was 105 ± 14 b.p.m. and persisted at the time of maximum vasovagal effect, although with a slight attenuation.
- These results support the beneficial additive effect of CLS on dual-chamber pacing in maintaining cardiac output and preventing syncopal recurrences.

## Introduction

Recent studies have shown that a dual-chamber pacemaker in closed-loop stimulation (CLS) rate-adaptive mode is highly effective in reducing syncopal recurrences and improving quality of life in patients with a tilt-induced asystolic vasovagal syncope (VVS).^1–4^ However, little is known about the operating mode of the CLS algorithm during VVS and exactly how it adds to the sole effect of dual-chamber pacing. It is well known that a compensatory mechanism counteracting vasodilation and pressure drop during the pre-syncopal phase of the vasovagal reflex leads to an increase in heart rate (HR) and ventricular contraction velocity, affecting intra-cardiac impedance. Since CLS is controlled by changes in intra-cardiac impedance, a possible increase in the CLS pacing rate (or the onset of CLS pacing above the intrinsic rate) during the pre-syncopal phase may alter haemodynamic response in a manner preventing the syncope.

To clarify this issue, we examined the temporal relationship between haemodynamic changes and CLS stimulation during a tilt-induced vasovagal reflex.

## Methods

Our prospective, non-randomized, acute tilt test (TT) study was conducted in the Syncope Units of the General Hospital in Bolzano and the Monaldi Hospital in Naples, Italy. The study protocol was approved by the local Ethics Committees.

The study included 20 patients with a dual-chamber CLS pacemaker indicated through a tilt-induced asystolic VVS. Seventeen of these patients had previously participated in the BioSync trial,^1^ and the other three received the pacemaker according to the same inclusion and exclusion criteria. In brief, patients had to be ≥40 years old, to have at least two episodes of unpredictable severe reflex syncope within a year before pacemaker implantation, and to have syncope inducible by a TT with an asystolic pause of >3 s. All patients provided written informed consent.

### Tilt testing

In the present study, the patients were asked to repeat the TT during late follow-up. Tilt tests were performed using a Task Force® device (CNSystems, Austria) and in accordance with the Italian protocol, which consisted of a 20 min tilt at 70° and (if negative) an additional 15 min tilt after a sublingual administration of 0.3 mg nitroglycerin.^5^ The test was terminated when syncope was induced or at the end of the tilt time. Electrocardiogram and blood pressure (BP) monitoring were recorded continuously throughout the test, and the whole test was video-recorded using an open-access video TT set-up as previously described.^6^ The video images were combined with the haemodynamic parameters of the Task Force monitor and used to record the exact time of onset of syncope (loss of facial, jaw, or neck tone; involuntary eye opening; or a lack of response).

During the TTs, all patients had the same standard pacemaker programming as in the BioSync trial:^1^ DDD-CLS mode with a basic rate of 50 b.p.m., the maximum CLS rate programmed at 120 b.p.m., CLS rate-adaptive response programmed at ‘Medium’, and resting rate control OFF.

### Outcome measures

The following haemodynamic parameters were used in the analysis: HR, systolic BP (SBP), diastolic BP (DBP), and pulse pressure (PP). Pulse pressure was calculated as SBP–DBP. Pacing in CLS mode was considered assessed when atrial-paced beats were consistently observed at a rate higher than the programmed basic rate (regardless of ventricular-sensed/paced beats). For ventricular-paced beats and atrial-sensed beats, CLS was considered unassessed.

Assessments of HR, SBP, DBP, and PP were made at rest before tilting up, at the time of onset of CLS atrial pacing (independently of sequential spontaneous or paced ventricular beat), at the time of the maximum CLS pacing rate, and at the time of syncope or the lowest SBP in patients without syncope. For each parameter, the result was calculated as the average for 10 consecutive beats.

### Statistical analysis

Continuous variables are reported as mean ± standard deviation or median and inter-quartile range (IQR) in case of non-normal distribution. Categorical variables are shown as absolute and relative frequencies. Changes in HR, SBP, DBP, and PP were investigated across four stages of the TT: baseline (Stage 1), pre-syncopal phase (Stage 2), maximum HR/pacing rate (Stage 3), and syncope or lowest SBP (Stage 4). Variations were assessed with linear-mixed models using HR, SBP, DBP, and BP as the dependent variables and the TT stages as fixed effects. Random intercepts were set at patient levels. Dependence on the TT stages was first investigated by including all stages in the model. Then, if significance was reached, prespecified comparisons were performed between consecutive stages (Stage 1 vs. 2, Stage 2 vs. 3, and Stage 3 vs. 4). *P*-values <0.05 were considered statistically significant after Bonferroni correction for multiple comparisons. Statistical analyses were performed with STATA/MP 18.0 (StataCorp, College Station, TX, USA).

## Results

A total of 20 patients had a mean age of 68.3 ± 11.9 years, and 16 (80%) were male. The baseline resting HR was 72 ± 6 b.p.m. Two patients had a first-degree atrioventricular block, four patients had a history of atrial fibrillation, and two patients had a history of coronary artery disease. The median ejection fraction was 56% (55–60). At baseline TT, all patients had syncope induced with an asystolic pause >3 s [median 6 s (IQR, 5–10)].

The patients were implanted with a dual-chamber CLS pacemaker at a median of 3.9 years before the present study (IQR, 1.5–5.0 years). Since pacemaker implantation, 19 (95%) of 20 patients remained free of syncopal recurrences, and one patient (5%) had a syncope preceded by prolonged prodromes while working at high altitudes and low temperatures. The percentages of pacing during follow-up were as follows: atrial pacing 46% (IQR, 36.8–50.2) and ventricular pacing 0.5% (IQR, 0.0–5.2).

The TT in the present study induced the pre-syncopal phase of a vasovagal reflex in 19 (95%) patients (all during the nitroglycerin phase), which ended with syncope in 11 (55%), was aborted in 8 (40%), and was negative in 1 patient. The latter patient was excluded from the final analysis along with two patients due to the violation of study protocol, as the ‘resting rate control’ parameter was not turned OFF but was set at 20 b.p.m., preventing the CLS pacing rate from increasing above 70 b.p.m.

### Haemodynamic changes and temporal relationship with closed-loop stimulation activation

**Figure 1:**
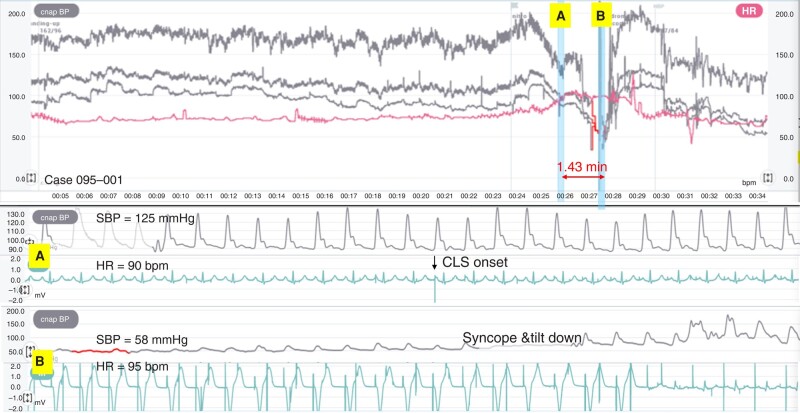
CLS pacing activated during a tilt test, followed by syncope. The top panel shows the trends of BP (grey lines: systolic, mean, and diastolic) and HR (red line) during the tilt test. After a stable phase, BP began to fall and HR began to rise from the 24th minute. Atrial-paced ventricular-sensed CLS pacing onset occurred after 26 min (blue vertical line A and bottom panel *A*). At that time, SBP and HR were 125 mmHg and 90 b.p.m.. Thereafter, SBP and HR briefly increased to 145 mmHg and 102 b.p.m. At 1.43 min after the onset of CLS pacing, the SBP rapidly fell to 58 mmHg, and the patient lost consciousness during sequential atrial and ventricular CLS pacing at 95 b.p.m. (blue vertical line B and bottom panel *B*). After tilting down, there was an immediate BP overshoot and a progressive decrease in HR. BP, blood pressure; CLS, closed-loop stimulation; HR, heart rate; SBP, systolic BP.

**Figure 2:**
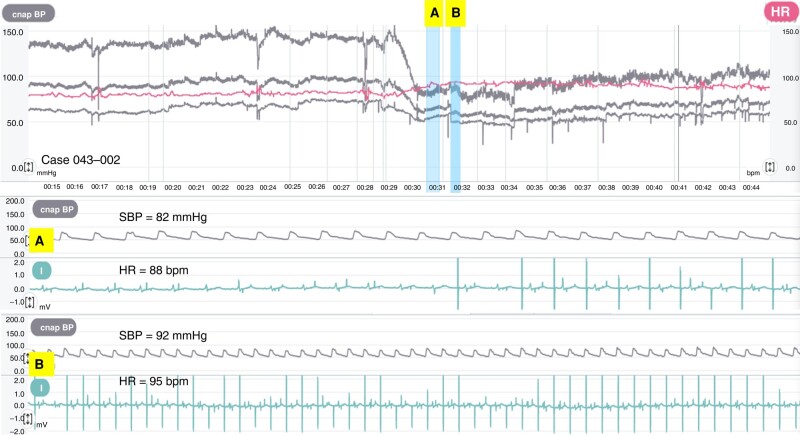
An impending symptomatic tilt-induced VVS, which was aborted during CLS pacing activation. The top panel shows the trend of BP (grey lines: systolic, mean, and diastolic) and HR (red line) during a tilt test. After a stabilization phase, BP began to fall from the 29th minute. Atrial-paced ventricular-sensed CLS pacing onset occurred after 30 min (blue vertical line A and bottom panel *A*). At that time, SBP and HR were 82 mmHg and 88 b.p.m. The atrial CLS pacing rate increased to 95 b.p.m. (blue vertical line B and bottom panel *B*) and remained stable until the 44th minute, when the test was stopped, and the patient was tilted down. The SBP varied slightly between 80 and 90 mmHg until the 34th minute, when the SBP exceeded 100 mmHg until the end of the test. BP, blood pressure; CLS, closed-loop stimulation; HR, heart rate; SBP, systolic blood pressure; VVS, vasovagal syncope.

The remaining 17 patients were included in the analysis of haemodynamic changes in relation to CLS activation. In 14 (82%) of 17 patients, CLS pacing occurred during the TT, and the other 3 patients (18%) had an intrinsic rhythm or basic rate pacing all the time. Two examples of CLS activation, one followed by a syncope and the other not followed by a syncope, are shown in *Figures 1* and *2*.

**Figure 3:**
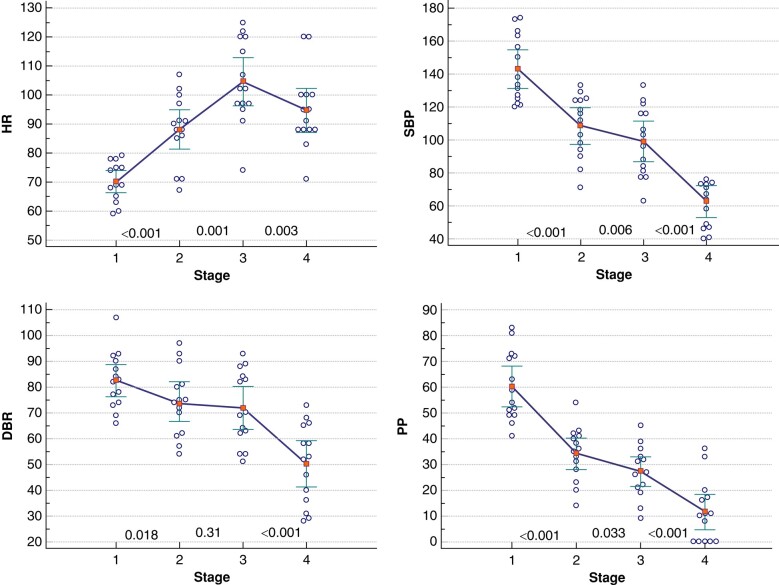
Haemodynamic changes during tilt tests in 14 patients with CLS pacing activation. Data are summarized as mean (red squares) and 95% confidence interval (whiskers). The circles indicate individual values. Stage 1 is the baseline period before tilting up. Stage 2 is the time of CLS pacing onset. Stage 3 is the period of the maximum CLS pacing rate. Stage 4 is the time of syncope or of the lowest SBP in patients without syncope. For each parameter, the result was calculated as the mean value for 10 consecutive beats. *P*-values refer to the comparison of two consecutive stages. CLS, closed-loop stimulation; DBP, diastolic blood pressure; HR, heart rate; PP, pulse pressure; SBP, systolic blood pressure.

**Figure 4:**
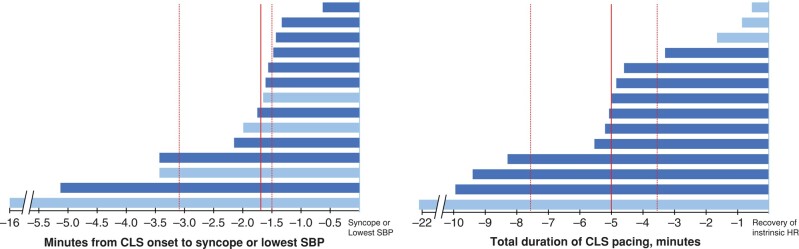
The individual duration of CLS pacing in 14 patients (1 bar per patient). The duration of CLS pacing from the CLS onset to syncope or the lowest SBP (left panel) and the total duration of CLS pacing from the CLS onset to the recovery of intrinsic sinus rhythm (right panel). Light blue bars represent 4 patients who did not experience syncope; dark blue bars represent 10 patients with syncope. The solid red line indicates median duration, and the dotted red lines indicate the inter-quartile range. The patients with syncope also had a longer CLS pacing, suggesting a longer duration of the vasovagal reflex. CLS, closed-loop stimulation; HR, heart rate; SBP, systolic blood pressure.

The 14 patients with CLS pacing had a baseline SBP of 143 ± 20 mmHg and a HR of 70 ± 7 b.p.m. (Stage 1 in *Figure 3*). CLS pacing emerged during the pre-syncopal phase of circulatory instability (Stage 2), when the intrinsic HR rose to 88 ± 12 b.p.m. and the SBP fell to 108 ± 19 mmHg (for both, *P* < 0.001 vs. Stage 1). Thereafter, the CLS pacing rate increased rapidly to a maximum of 105 ± 14 b.p.m. within a median of 0.1 min (IQR, 0.1–0.7 min) when the SBP was 99 ± 21 mmHg (Stage 3). At the time of maximum vasovagal effect, the CLS pacing rate decreased to 95 ± 13 b.p.m. and the SBP was 63 ± 17 mmHg, followed by syncope in 10 patients (Stage 4 in *Figure 3*). Diastolic blood pressure and PP showed similar patterns to SBP. The onset of CLS pacing was 1.7 min before syncope or lowest SBP (IQR, 1.5–3.4 min; *Figure 4*). The total duration of CLS pacing from onset to recovery of an intrinsic HR after tilting down was 5.0 min (IQR, 3.3–8.3 min; *Figure 4*).

The three patients without CLS pacing during the TT had a competing intrinsic rate and a basic pacing rate of around 50 b.p.m., with one of these experiencing syncope. Compared with patients with CLS pacing, the patients without CLS pacing had a similar SBP decrease from 142 ± 23 mmHg at baseline to 69 ± 4 mmHg at the time of maximum vasovagal effect, but there was no significant increase in HR (59 ± 1 b.p.m.).

## Discussion

The main findings of this study are that the reproducibility of the pre-syncopal phase of a vasovagal reflex during the TT was high and that high-rate CLS pacing occurred in most patients early during the pre-syncopal phase of circulatory instability, to persist, although attenuated, at the time of maximum vasovagal effect.

These findings are important and may help explain the positive results of the BioSync trial. The objective of cardiac pacing is to avoid vagally induced bradycardia. Because stroke volume and HR similarly contribute to determining BP and cardiac output at the time of impending syncope, an increase in HR achieved by CLS early during the pre-syncopal phase (Stage 2) could sustain cardiac output even when BP falls rapidly due to the vasodilation reflex.^7^ The activation of CLS on a median of 1.7 min before the time of maximum vasovagal effect when BP was still high to maintain a sufficient cerebral blood flow and the increase in HR to a maximum rate of 105 b.p.m. suggest that CLS may have a beneficial additive effect to that of dual-chamber pacing in maintaining cardiac output and preventing syncopal recurrences. All patients included in this study, except one, can be defined as clinical responders because they remained free of syncopal recurrences after a median of 3.9 years of pacemaker implantation. The fact that 40% of patients ended with syncope during the TT should not be misleading here, because a TT is known to be an unreliable test for demonstrating the effectiveness of pacing therapy in clinical settings.^8–10^ Similar effects on HR and SBP were already observed by Palmisano *et al.*^11^ and in an ancillary substudy of the BioSync trial.^12^ In a randomized, cross-over study,^11^ DDD-CLS pacing reduced the occurrence of syncope induced by a TT, compared with DDD pacing. A plausible reason is that, during a TT, syncope occurs while the patient stands in the fixed upright position, which is forced by the tilt table without the possibility of activating countermeasures and interrupting progression towards loss of consciousness. Conversely, during spontaneous episodes, patients may react against reflex and hypotension, assuming sitting or supine positions at symptom onset. In this situation, hypotension is usually less severe and insufficient to cause a complete loss of consciousness.^10^

**Figure 5:**
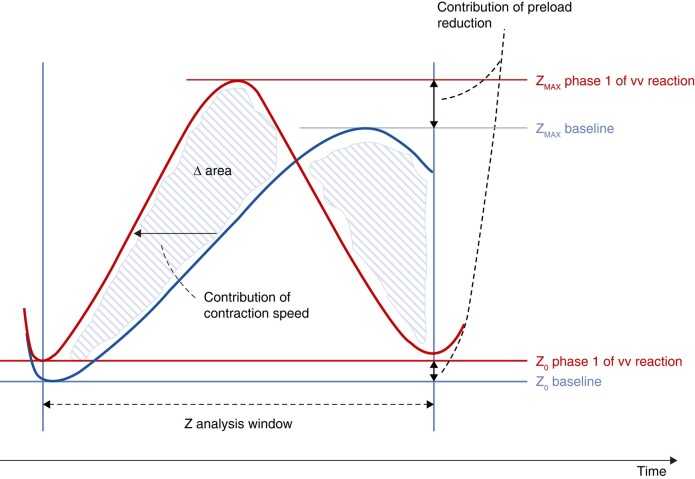
The mechanism of CLS. The CLS algorithm measures intra-cardiac impedance (*Z*) curves of the right ventricle during the systolic phase of each cardiac cycle by injecting sub-threshold high-frequency unipolar current pulses from the ventricular lead tip. Given that the density of electric field lines is greatest around the electrode tip, most of the induced voltage drop at each sub-threshold pulse and, in turn, the main contribution to impedance, occurs in an ∼5 cm^3^ volume (≍1 cm radius) surrounding the tip electrode. The local changes in the amount of blood and myocardial tissue in the small volume surrounding the tip electrode are the main drivers of measured impedance variations, rather than the changes in the entire intra-ventricular volume. During contraction, blood ejection causes tighter contact of the electrode with myocardial tissue (involving a larger proportion of tissue relative to blood volume), leading to a progressive impedance increase towards late systole. The more the speed of contraction increases, the more the slope of the impedance curve becomes steep (red curve) in comparison with a reference impedance curve obtained during patient resting periods (blue curve). The difference in the area between the two curves (Δarea) determines the increase in pacing rate. The main contribution to Δarea may be assumed to be related to the increased contraction speed during the pre-syncopal phase, while a minor contribution may be ascribed to the progressive reduction in preload (reduced filling) in this phase causing an increase of minimum (*Z*_MIN_) and maximum (*Z*_MAX_) peaks of impedance curves during vasovagal reaction. See also Tomaino *et al.*^10^ CLS, closed-loop stimulation.

We observed a great variability of CLS responses during the TTs. The maximum CLS pacing rate ranged from 74 to 120 b.p.m. among patients who had CLS pacing during the TTs, whereas three other patients had no onset of CLS pacing, probably representing the low extreme of such variability. This variability requires some tentative explanations. There are two possible complementary reasons. Given that the CLS algorithm continuously adjusts the pacing rate in response to differences detected in electrode–tissue impedance trends during the systolic phase of each cardiac cycle (*Figure 5*), the first reason may be related to the inter-patient variability of impedance trends, e.g. due to individual electrical characteristics at the electrode–tissue interface. The second reason may be different pathophysiologies of the vasovagal response from patient to patient, even when the clinical features are similar. Since the CLS algorithm continuously monitors impedance trends during systoles, the changes in the CLS pacing rate can be expected to correspond primarily to changes in the speed of cardiac contraction during systole (*Figure 5*),^13,14^ thus reflecting different inter-patient magnitudes of baroreflex compensatory mechanisms in response to orthostatic stress (see section below). In the extreme case, the lack of HR increase in three patients who did not have an onset of CLS pacing suggests the absence of compensatory mechanisms as observed in patients with orthostatic hypotension. If this explanation is true, CLS could be intended as a tool to measure baroreflex activity during VVS. However, this variability has practical clinical implications (see the *Clinical implications and suggestions for potential improvements to the closed-loop stimulation algorithm in the reflex syncope applications* section).

### Temporal correlation between the pathophysiology of vasovagal syncope and closed-loop stimulation behaviour

In accordance with the classification of the haemodynamic evolution of tilt-induced VVS recently proposed by Jardine *et al*.^15^ and van Dijk *et al*.,^7^ for the purpose of studying the CLS mechanism, we consider the following phases of a TT.

#### Pre-syncopal phase (circulatory instability)

During this phase, tilt-positive patients show an increase in HR, a mild gradual decrease in SBP and DBP with increased BP variability, and a mild gradual decrease in stroke volume (the large decrease in PP observed in the present study can be considered as an indirect estimate of stroke volume) and in total vascular resistances.^7,15^ These haemodynamic changes are the expression of compensatory mechanisms in response to orthostatic stress. Heart rate increases due to baroreceptor activation and neuroendocrine response to counteract the decrease in stroke volume caused by venous pooling in the abdomen, pelvis, and skeletal muscles.

**Figure 6:**
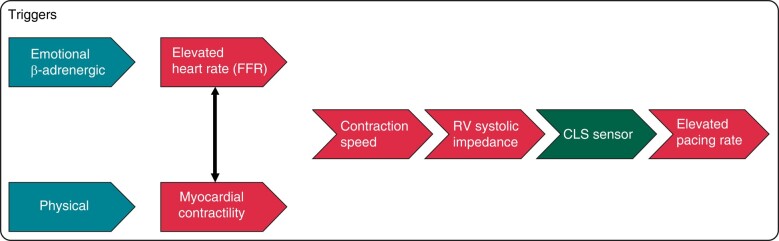
Factors triggering CLS and pacing rate adaptation. When reading the scheme from right to left, the pacing rate is increased by CLS, which is triggered by a detected variation of the shape of rising impedance curves during systole. Changes in RV impedance are strictly correlated to RV dP/dt max, which is a surrogate of RV contraction speed. Contraction speed in turn is influenced by two factors: elevated heart rate and myocardial contractility. Reprinted with permission from Tomaino *et al*.^10^ CLS, closed-loop stimulation; FFR, force–frequency relationship; RV, right ventricular.

At some point in this phase, the CLS pacing rate exceeds intrinsic HR. During CLS pacing, the slopes of SBP, DBP, and PP decrease, suggesting a partial effect of HR increase to counteract the decrease in stroke volume (*Figure 2*). In the present study, the end of the pre-syncopal phase coincides with the time of maximum increase in the CLS pacing rate. Although the operating principle of CLS is sufficiently clear to explain its original use as rate-responsive pacing triggered by the strength of myocardial contractility during physical activity in patients with chronotropic incompetence,^16^ the CLS activation during the pre-syncopal phase of VVS remains a matter of investigation because contractility is unlikely to come into play at this stage, given that stroke volume decreases and the Frank–Starling principle cannot be invoked.^7^ The increase in intrinsic HR resulting from baroreceptor activation and the neuroendocrine response to counteract vasodilation is *per se* capable of increasing the intra-cardiac impedance detected by the CLS algorithm. This is a physiological phenomenon known as positive force–frequency relationship (FFR). Under normal conditions, up to 40% of cardiac output is regulated through the positive FFR principle.^17^ The FFR is influenced by β-adrenergic regulation, which increases during the pre-syncopal phase and in other conditions such as active standing, handgrip, cold pressor test, mental stress, and dobutamine infusion (*Figure 6*).^14,17–21^

#### Syncopal phase (cardioinhibition)

This phase begins when HR starts to decrease due to the activation of the vagal reflex and ends at syncope epoch,^7^ accompanied by a rapid fall in BP.^15^ The onset of cardioinhibition is both a fundamental and a literal turning point. During this phase, even a modest decrease in HR is associated with an immediate acceleration of the fall in BP until it reaches values critical for maintaining sufficient cerebral blood flow. Reduction of stroke volume and total peripheral resistance is of minor relevance.

During the syncopal phase, the CLS pacing rate decreases. We can suppose that the reason for this decrease is related to the termination of the same factors that caused the pacing rate increase during the pre-syncopal phase. Even if minor, a reduction in the CLS pacing rate is counterproductive because it occurs when there is a maximum need for a high HR to maintain stroke volume and cardiac output. However, even if sub-optimal, the observed CLS pacing rate of 95 ± 13 b.p.m. at the time of maximum vasovagal effect explains why CLS pacing delayed the onset of syncope during the TT compared with conventional dual-chamber pacing in an intra-patient comparison study^11^ and may be the root cause of CLS effectiveness in preventing clinical syncope in the BioSync trial.^1^

### Clinical implications and suggestions for potential improvements to the closed-loop stimulation algorithm in reflex syncope applications

Due to the large variability of CLS responses, which cannot be foreseen at the time of pacemaker implantation, it is likely that the standard empirical programming of the CLS function used in the BioSync trial^1^ and in the present study was not adequate in some patients. In a study by Prakash *et al.*,^22^ 13 patients had spontaneous syncope or pre-syncope after CLS pacemaker implantation and a positive vasodepressor tilt response. They repeated tilt testing with the CLS response reprogrammed to a steeper rate response (‘High’ level), and 7 (54%) of them had no symptoms (had a negative TT). In addition, the recorded impedance information was downloaded from the pacemakers and processed offline by the CLS algorithm. It was calculated that the CLS response programmed to ‘High’ would have resulted in a pacing rate increase of 20–50 b.p.m. (mean, 21 ± 11 b.p.m.). Thus, a post-implant TT may be useful to achieve personalized programming in patients who had syncopal recurrences after pacemaker implantation.

The results of the present study portend some potential improvements for a ‘syncope-specific’ version of CLS in the future. First, the slight decrease in the CLS pacing rate observed at the time of maximum vasovagal effect should be best avoided by maintaining the maximum CLS pacing rate throughout the duration of the syncopal phase. Based on the present data, CLS pacing should last some minutes longer than an average of 1.7 min observed in our study and then progressively decay to a basic rate. Programmability of the duration of the maximum rate is advisable.

Second, the pacemakers used in this study provide intra-cardiac electrogram recordings for high atrial and ventricular rate episodes, but not for suspected syncopal or pre-syncopal episodes. This is understandable as CLS was designed to be an advanced rate-responsive system and a pacemaker certainly cannot detect impending loss of consciousness. Nevertheless, in a ‘syncope-specific’ version of CLS, it would be desirable to also record snapshots of intra-cardiac impedance or some related parameters during periods of high-rate atrial pacing (which are rare in these patients^23^) to assess episodes and guide device programming. The combination of impedance information with activity and body position sensors and patient-activated symptom information would likely help distinguish impending reflex syncope from other situations of CLS activation.

Third, artificial intelligence and machine learning algorithms offer new perspectives in identifying specific activation patterns to enable the detection of impending VVS before loss of consciousness. In this way, the CLS algorithm may become a diagnostic tool used to warn the patient to take preventive measures.

## Conclusions

The reproducibility rate of a vasovagal reflex was as high as 95%, higher than usually assumed based on old studies in the literature.^24^ Closed-loop stimulation pacing was observed in most patients early in the pre-syncopal phase and persisted at the time of maximum vasovagal effect, although with a slight attenuation. These results support the beneficial additive effect of CLS on dual-chamber pacing in maintaining cardiac output and preventing syncopal recurrences.

## Data Availability

The data underlying this article will be shared on reasonable request to A.G., Research Clinical Unit, Biotronik Italy, Italy. Email: alessio.gargaro@biotronik.com.
